# Arteriovenous malformations of the posterior fossa: surgical excellence and technological innovation

**DOI:** 10.3171/2020.10.FOCVID20105

**Published:** 2021-01-01

**Authors:** Mustafa K. Baskaya, Adib A. Abla, Daniel L. Barrow, Adam S. Arthur

**Affiliations:** 1 Department of Neurological Surgery, University of Wisconsin–Madison, Wisconsin;; 2 Department of Neurological Surgery, University of California, San Francisco, California;; 3 Department of Neurosurgery, Emory University School of Medicine, Atlanta, Georgia; and; 4 Department of Neurosurgery, Semmes-Murphey Neurologic and Spine Institute, Memphis, Tennessee

Arteriovenous malformations (AVMs) of the posterior fossa are less common than their supratentorial counterparts but have higher rates of rupture along with higher morbidity and mortality. Treatment of these lesions is difficult due to the complexity of the posterior circulation, with frequent anatomic variations and perforators supplying the brainstem. Microsurgical resection can be challenging, with the potential need for complex surgical approaches with limited access to the lesion through narrow working corridors. We are honored to have been selected to edit this important issue of *Neurosurgical Focus: Video*. We have selected 15 videos from four continents. These videos were chosen for their demonstration of state-of-the-art techniques in the treatment of posterior fossa AVMs via microsurgical resection. This collection of videos by masters in the field of neurovascular surgery illustrates surgical decision-making, technical mastery, operative nuances, technological innovations, and complication avoidance, demonstrating the beauty and danger of AVMs in the posterior fossa location.

It is our hope that this collection of videos serves as an educational tool for neurosurgical trainees in understanding these lesions, while also providing an update on the current state of surgical treatments for more experienced clinicians. This compendium shows novel vascular treatment concepts, innovative approaches, the evolution of intraoperative imaging, and advanced technologies. While these new techniques expand the options for treating posterior fossa AVMs, microsurgical excellence founded in a detailed understanding of neuroanatomy forms the basis of the surgical mastery apparent in these videos. We thank all the authors for their excellent contributions.

